# Dynamic graph based attention spectral network for motor imagery-brain computer interface

**DOI:** 10.3389/fnhum.2026.1755549

**Published:** 2026-03-04

**Authors:** Zexiong Shao, Zhenghui Gu, Le Che, Zhuliang Yu, Yuanqing Li

**Affiliations:** 1 The School of Automation Science and Engineering, South China University of Technology, Guangzhou, China; 2 The Institute for Super Robotics (Huangpu), Guangzhou, China; 3 The Pazhou Laboratory, Guangzhou, China; 4 The School of Architecture, South China University of Technology, Guangzhou, China; 5 Shien-Ming Wu School of Intelligent Engineering, South China University of Technology, Guangzhou, China

**Keywords:** brain-computer interface (BCI), convolution neural network (CNN), cross-spectro interaction, electroencephalogram (EEG), graph neural network (GNN), motor imagery (MI)

## Abstract

Motor imagery-based brain computer interface (MI-BCI) have been increasingly adopted in neurorehabilitation and related fields. The performance of MI-electroencephalogram (MI-EEG) decoding algorithms is central to the advancement of MI-BCI. However, current studies often lack rigorous investigation into the brain's complex network organization. Moreover, most existing methods do not incorporate the cross-frequency coupling (CFC) phenomena that occur during MI into their algorithmic designs, nor do they adequately account for how temporal dynamics across different MI stages influence decoding outcomes. To address these limitations, we propose the Dynamic Spectral-Spatial Interaction Convolution Neural Network (DSSICNN), a parameter-efficient MI-EEG decoding framework that jointly extracts temporal-spectral-spatial features. DSSICNN adopts a dual-branch parallel architecture to concurrently learn spatial representations in both Euclidean and non-Euclidean domains. It further integrates a CFC-inspired attention module to model cross-spectral interactions, followed by an additional attention mechanism that quantifies the contributions of distinct MI stages to decoding performance. DSSICNN achieves decoding performance on two public datasets that surpasses the current state-of-the-art (SOTA) under both session-dependent and session-independent settings. Beyond its empirical advantages, DSSICNN offers design insights for developing Graph Neural Network (GNN)-based MI-EEG decoding algorithms and provides a network neuroscience-inspired perspective for understanding the neurophysiological mechanisms underlying MI.

## 1 Introduction

Brain computer interfaces (BCI) constitute an emerging framework for human machine communication by converting neural activity directly into control signals for external systems (Edelman et al., 2025). Among the available neural sensing modalities, electroencephalography (EEG) has gained substantial prominence owing to its non-invasiveness, affordability, and excellent temporal resolution. Motor imagery (MI), defined as the internal simulation of voluntary limb movements in the absence of overt motor output, induces characteristic modulations in the sensorimotor cortex, particularly within the mu (8–12 Hz) and beta (18–26 Hz) frequency bands, observed as event-related synchronization/event-related desynchronization (ERS/ERD). Because MI enables users to initiate control voluntarily without depending on external cues, it has become a focal point of contemporary research. MI-EEG-BCI have been applied across a wide range of clinical and rehabilitative contexts (AL-Quraishi et al., 2018). Consequently, the effectiveness of MI EEG decoding strategies is central to the overall reliability and utility of MI-BCI.

MI-EEG decoding techniques are generally grouped into two major classes: machine learning (ML) algorithms and deep learning (DL) algorithms. ML algorithms derive discriminative representations from MI-EEG data by quantifying signal characteristics in temporal, spectral, and spatial domains. Notable examples include Filter Bank Common Spatial Patterns (FBCSP) (Ang et al., 2008) and the Continuous Wavelet Transform (CWT) (Hsu and Sun, 2009). Although widely adopted, these ML-based strategies rely substantially on handcrafted feature engineering, which demands considerable neurophysiological expertise. Such dependence limits their capacity to achieve further gains in decoding accuracy (Altaheri et al., 2023).

In recent years, the rapid advancement of deep learning (DL) has encouraged the exploration of DL-based strategies for MI-EEG decoding (Wang X. et al., 2023; Bhambare and Jain, 2024; Gu et al., 2025), yielding substantial improvements. DL models support end-to-end representation learning, enabling the automatic derivation of informative latent features directly from raw EEG signals. As a result, DL-driven decoding frameworks mitigate the need for extensive neurophysiological domain expertise. Prominent examples include Shallow ConvNet and Deep ConvNet (Schirrmeister et al., 2017), as well as EEGNet (Lawhern et al., 2018) and FBCNet (Mane et al., 2021).

In recent years, graph neural network (GNN) have attracted increasing attention for EEG decoding, motivated by the need to more effectively characterize the spatial properties of EEG signals (Song et al., 2020; Du et al., 2022; Feng et al., 2022). By representing EEG recordings as graph structured data, GNN account for signals from electrodes and offer a more expressive modeling of spatial heterogeneity than traditional convolution based techniques. Despite these advantages, existing GNN driven MI-EEG decoding approaches encounter several persistent limitations. First, adjacency matrices derived from predefined similarity measures may inadequately represent functional relationships among cortical regions, potentially omitting salient neural interaction patterns. When the adjacency structure is learned, the number of parameters grows quadratically with the number of electrodes, resulting in substantial computational overhead. In addition, these adjacency matrices are typically fixed during inference, even though MI tasks may induce dynamic changes in functional connectivity, thereby constraining the model's ability to capture task specific variations. Furthermore, many current frameworks incorporate GNN modules directly into the decoding pipeline, and the computational burden associated with GNN operations further amplifies parameter growth. Finally, variations in cognitive states, such as mental fatigue, can influence the relative contributions of different experimental sessions to overall decoding performance, yet this factor is frequently disregarded in contemporary models.

To address the aforementioned limitations, we propose Dynamic Spectral-Spatial Interaction Convolution Neural Network (DSSICNN). The primary contributions of this work are summarized as follows:

- We propose a dual-branch parallel framework constructed to concurrently derive spatial semantic features associated with MI.
- To account for the differential contributions of distinct phases within MI process to decoding outcomes, we introduce an attention-guided module for spectrally informed temporal aggregation.
- We performed extensive comparative evaluations to assess the performance of DSSICNN. The findings indicate that DSSICNN achieves substantial improvements over state-of-the-art (SOTA).

## 2 Related work

### 2.1 Attention mechanism

Over the past decade, attention mechanisms have experienced significant advancements. SENet (Hu et al., 2018) introduced the principle of channel-wise attention, which has subsequently influenced the development of numerous architectures, including *A*^2^-Net (Chen et al., 2018), CBAM (Woo et al., 2018), ECANet (Wang et al., 2020), and FcaNet (Qin et al., 2021). These mechanisms have also been extensively applied in MI-EEG classification (Song et al., 2023), where their integration into decoding frameworks frequently results in marked performance enhancements. Inspired by these developments, we integrate attention mechanisms within DSSICNN.

### 2.2 Cross-frequency coupling

CFC denotes the modulation of neural oscillatory activity in one frequency band by activity in another band. Empirical studies have demonstrated that during MI tasks, CFC occurs across multiple frequency bands (Feng et al., 2020). Moreover, distinct MI tasks induce characteristic CFC patterns across both spectral and spatial dimensions. Consequently, CFC constitutes a vital source of discriminative features relevant to MI decoding.

## 3 Methods

**Figure 1:**
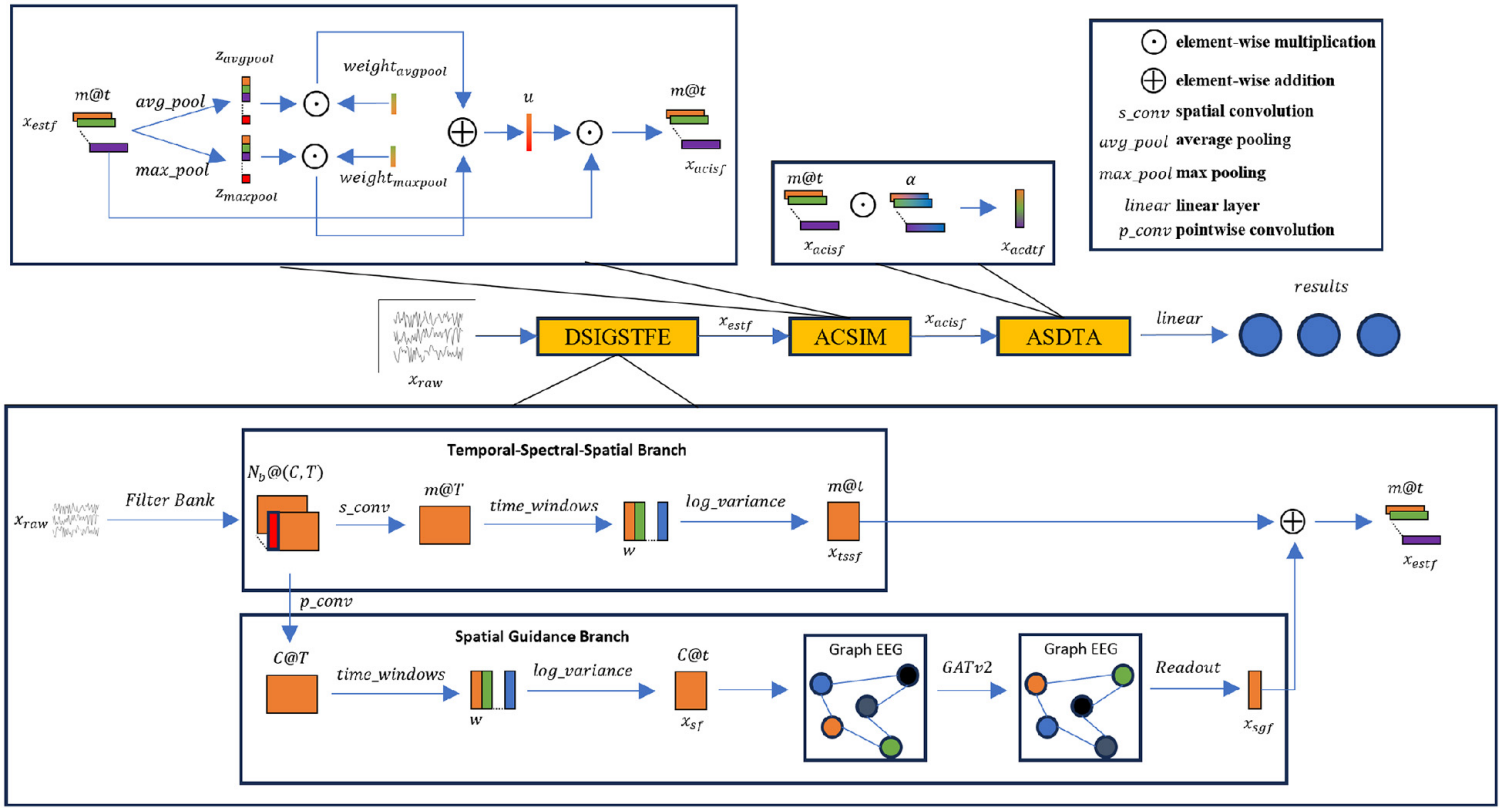
The overall architecture of DSSICNN.

The detailed architecture of DSSICNN is illustrated in Figure 1. DSSICNN mainly consists of three components: Dynamic Spatial Interaction Graph-guided Spatio-Temporal Feature Extraction (DSIGSTFE), which comprises Temporal-Spectral-Spatial Branch and Spatial Guidance Branch; Attentive Cross-Spectro Interaction Module (ACSIM); and Attentive Spectro-Dependent Temporal Aggregation (ASDTA).

### 3.1 Two forms of EEG

#### 3.1.1 Multi-view EEG

Owing to inter-subject variability in the frequency bands associated with ERD/ERS, decomposing EEG signals into multiple sub-bands via band-pass filter banks has been shown to improve MI-EEG decoding performance (Ang et al., 2008; Mane et al., 2021; Liu et al., 2023). Specifically, let $x_{raw}\in R^{C\times T}$ represent the raw EEG signal, where *C* denotes the number of scalp electrodes and *T* denotes the number of temporal samples. The raw MI-EEG data are then decomposed into $x_{fb}\in R^{N_{b}\times C\times T}$ using a band-pass filter bank, where *N*_*b*_ corresponds to the number of filters applied. The band-pass filter bank consists of nine band-pass filters with frequency ranges of 4–8 Hz, 8–12 Hz, 12–16 Hz, 16–20 Hz, 20–24 Hz, 24–28 Hz, 28–32 Hz, 32–36 Hz, and 36–40 Hz, respectively.

#### 3.1.2 Graph EEG

As described in Wu et al. (2021), a graph can be formally defined as *G* = (*V, E, A, H*), where *V* represents the set of nodes, *E* the set of edges, *A* the adjacency matrix, and *H* the node feature matrix. In the context of EEG, nodes correspond to scalp electrodes, while edges and the adjacency matrix capture the functional or structural connectivity between the brain regions associated with electrode pairs. The matrix *H* encodes the features corresponding to each EEG electrode.

EEG-derived brain networks frequently include spurious connections arising from noise, which can interfere with inter-regional information transfer and impair MI-EEG decoding performance. To mitigate this issue, spurious links are reduced by estimating functional connectivity (FC) between brain regions, which measures their statistical interdependencies. In the present study, FC is evaluated using the phase locking value (PLV) (Lachaux et al., 1999), and the resulting PLV measurements are utilized to construct the adjacency matrix based on the following criteria:


$$\begin{array}{l} PLV_{ij}=\frac{1}{T}|\overset{T}{\underset{t=1}{\sum }}e^{i\left(\varphi_{i}\left[t\right]-\varphi_{j}\left[t\right]\right)}| \end{array}$$ (1)

$$\begin{array}{l} A_{ij}=\{\begin{array}{c} 1,\;PLV_{ij}>threshold\\ 0,\;PLV_{ij}<threshold \end{array} \end{array}$$ (2)

here, *PLV*_*ij*_ denotes the PLV between the signals of the *i*^*th*^ and *j*^*th*^ EEG electrodes and φ[*t*] denotes the phase of the analytic signal, which is derived from *x*_*raw*_ through the Hilbert transform. To facilitate model convergence and ensure consistent graph sparsity, the threshold is set to the 100^*th*^ largest PLV value.

In summary, FC between brain regions is quantified using the PLV. Connections exhibiting lower PLV values, which are regarded as spurious links introduced by noise, are subsequently removed (Klepl et al., 2022).

### 3.2 Dynamic spatial interaction graph-guided spatio-temporal feature extraction

The core concept of DSIGSTFE is to incorporate principles from network neuroscience (Bassett and Sporns, 2017) into the MI-EEG decoding pipeline to improve the extraction of spatial semantic features. Specifically, DSIGSTFE captures spatial features across both Euclidean and non-Euclidean domains simultaneously. While numerous studies (Liu et al., 2023) have utilized spatial convolutions to extract spatial information with considerable decoding accuracy, insights from network neuroscience indicate that EEG signals possess graph-structured characteristics, and spatial convolution alone is insufficient to comprehensively model interactions among brain regions, often referred to as spatial interactions. To address this limitation, a dynamic GNN is employed to model these spatial interactions and extract Spatial Guidance Feature, thereby enhancing the spatial representations derived from convolutional operations. Furthermore, in comparison with previous approaches, the proposed dynamic GNN is integrated into a parameter-efficient MI-EEG decoding framework, resulting in a novel architecture that demonstrates notable strengths in both methodological innovation and computational efficiency. The parameter-efficient MI-EEG decoding framework we designed effectively mitigates overfitting during training, while simultaneously ensuring that DSSICNN possesses strong potential for deployment in MI-BCI.

#### 3.2.1 Temporal-spectral-spatial branch

A spatial convolutional layer comprising *m* spatial filters with a receptive field of (*C*, 1) is utilized to extract spatial features. Given that the input consists of multi-band signals, information is concurrently aggregated across the spectral dimension, facilitating the extraction of spectral features. To map the input signals into a latent space with increased representational capacity and to enhance the spectral resolution of the resulting features for subsequent ACSIM processing, the condition *m*>*N*_*b*_ is imposed. Following the spatial convolution operation, a Batch Normalization (BN) layer (Ioffe and Szegedy, 2015) and an Exponential Linear Unit (ELU) activation function (Clevert et al., 2015) are applied. Additionally, the associated parameters are regularized by constraining the maximum L2 norm of the filter weights in the spatial convolutional layers.

In summary, the computation for the spatial convolutional layer is given by the following formulation:


$$\begin{array}{l} x_{ssf}=\sigma \left(F_{bn}\left(F_{conv}\left(x_{fb}\right)\right)\right) \end{array}$$ (3)

where *F*_*conv*_(·) is the spatial convolution layer, *F*_*bn*_(·) is the BN layer, σ(·) is the ELU activation function, $x_{fb}\in R^{N_{b}\times C\times T}$ is the multi-view EEG, and the $x_{ssf}\in R^{m\times T}$ is the output feature of the spatial convolution layer.

During MI trials, participants inevitably experience mental fatigue (Jacquet et al., 2021), which can lead to substantial fluctuations in MI-related temporal patterns over the course of a trial. To mitigate this effect, each trial is divided into non-overlapping temporal segments, and temporal features are extracted from each segment. Although convolution neural network (CNN) are frequently employed for temporal feature extraction, prior studies in electrophysiology and nonlinear dynamical neuroscience have shown that EEG signals are inherently non-stationary, limiting the capacity of CNN to fully capture temporal dynamics (Izhikevich, 2007). Therefore, CNN is not applied for temporal feature extraction in this study.

Motivated by the approach in Mane et al. (2021), a temporal log-variance layer is employed to extract temporal features from each time window. Specifically, *x*_*ssf*_ was partitioned along the temporal dimension using non-overlapping time windows of length *w*, and the logarithmic variance was computed for each time window. The computation of the temporal log-variance layer is given as follows:


$$\begin{array}{l} x_{tssf}=\sigma \left(F_{var}\left(x_{ssf}\right)\right) \end{array}$$ (4)

$$\begin{array}{l} F_{var}\left(x_{ssf}\right)\left[i,j\right]=\frac{1}{w}\overset{w\times \left(j+1\right)}{\underset{t=w\times j}{\sum }}{\left(x_{ssf}\left[i,t\right]-\mu \left[i,j\right]\right)}^{2} \end{array}$$ (5)

where $x_{tssf}\in R^{m\times t}$ represents the output feature of the temporal-spectral-spatial branch, $t=\left\lfloor \frac{T}{w}\right\rfloor$ represents the number of time window, σ(·) represents the logarithmic function, *F*_*var*_(·) represents the temporal log-variance layer, $x_{ssf}\in R^{m\times T}$ represents the output feature of the spatial convolution layer, *i* represents the *i*^*th*^ feature map, *j* represents the *j*^*th*^ time window, and μ[*i, j*] represents the mean of the *j*^*th*^ time window within the *i*^*th*^ feature map.

#### 3.2.2 Spatial guidance branch

Within the Spatial Guidance Branch, a dynamic GNN is utilized to generate spatial guidance features, which are subsequently employed to enhance the representations produced by the Temporal-Spectral-Spatial Branch. To ensure that these spatial guidance features effectively complement the outputs of the Temporal-Spectral-Spatial Branch, temporal alignment between the two branches is maintained. This is accomplished by first fusing the multi-spectral features from multi-view EEG using a (1, 1) pointwise convolution (Chollet, 2017). The fused features are then processed through a temporal log-variance layer, calculated as follows:


$$\begin{array}{l} x_{sf}=\phi \left(F_{var}\left(\sigma \left(F_{bn}\left(F_{conv}\left(x_{fb}\right)\right)\right)\right)\right) \end{array}$$ (6)

where $x_{sf}\in R^{C\times t}$ is the spectral-fused feature and serves as the node feature matrix used as input to the subsequent dynamic GNN, $x_{fb}\in R^{N_{b}\times C\times T}$ is the multi-view EEG, *F*_*conv*_(·) is the (1, 1) pointwise convolution, *F*_*bn*_(·) is the BN layer, σ(·) is the ELU activation function, *F*_*var*_(·) is the temporal log-variance layer, and ϕ(·) is the logarithmic function.

Subsequently, a dynamic GNN is employed to model interactions among brain regions. In this study, GATv2 (Brody et al., 2022) is adopted as the dynamic GNN, with its design principles largely derived from the original GAT architecture (Velicković et al., 2018). GATv2 is a convolutional-style GNN that leverages a self-attention mechanism, enabling the dynamic aggregation of node features by adaptively adjusting attention weights based on the properties of each node and its neighbors. Since its feature aggregation relies exclusively on local node-neighborhood relationships, GATv2 is well suited for applications where the underlying graph structure is incomplete or partially unknown. As noted in Section 3.1.2, accurately estimating connectivity using predefined statistical measures is challenging, often resulting in incomplete structural information in graph-based EEG representations. Therefore, GATv2 is particularly appropriate for integration into DSSICNN. The computational procedure of GATv2 is as follows:


$$\begin{array}{l} \vec{h_{i}^{\prime }}=\sigma \left(\frac{1}{N_{heads}}\overset{N_{heads}}{\underset{k=1}{\sum }}\underset{j\in N_{i}}{\sum }\alpha_{ij}^{k}{\text{W}}^{k}\vec{h_{j}}\right) \end{array}$$ (7)

where $\vec{h_{i}^{\prime }}\in R^{t}$ indicates the output feature of node *i*, σ(·) indicates the activation function, *N*_*heads*_ indicates the number of attention heads [defined similarly to Transformer (Vaswani et al., 2017)], *N*_*i*_ indicates the neighborhood of node *i*, $\alpha_{ij}^{k}$ indicates the normalized attention coefficients (computed based on the node features of node *i* and node *j*), **W**^*k*^∈*R*^*t*×*t*^ indicates the learnable linear transformation, and $\vec{h_{j}}\in R^{t}$ indicates the input feature of node *j*.

Once the node-level features are obtained, a readout function is necessary to generate a graph-level embedding, since the task entails classifying EEG signals from individual MI trials (i.e., graph classification). In this work, global sum pooling is employed as the readout function, as previous studies (Xu et al., 2019) have shown that it frequently yields more discriminative graph embeddings.

In summary, GATv2, in conjunction with global sum pooling, is applied to the spectrally fused features to generate the spatial guidance representations.


$$\begin{array}{l} x_{sgf}=F_{readout}\left(\sigma \left(F_{bn}\left(F_{gatv2}\left(G\right)\right)\right)\right) \end{array}$$ (8)

where $x_{sgf}\in R^{t}$ is the spatial guidance feature, *F*_*readout*_(·) is the global sum pooling, σ(·) is the ELU activation function, *F*_*bn*_(·) is the BN layer, *F*_*gatv*2_(·) is the GATv2 layer, and *G* = (*V, E, A, H*) is the graph EEG.

#### 3.2.3 Enhancing spatial domain representation using spatial guidance features

Taking into account the temporal and spectral compatibility of *x*_*tssf*_ and *x*_*sgf*_, as well as the need to control parameter complexity, a simple element-wise addition is utilized to augment the spatial representation within the overall model, as shown below:


$$\begin{array}{l} x_{estf}=x_{tssf}+x_{sgf} \end{array}$$ (9)

where $x_{estf}\in R^{m\times t}$ represents the enhanced spatio-temporal feature. Since *x*_*sgf*_ was derived from analyses grounded in network neuroscience, this process effectively integrates network neuroscience prior into MI-EEG decoding framework.

### 3.3 Attentive cross-spectro interaction module

At present, most DL-based MI-EEG decoding algorithms tend to introduce, in the shallow layers of the network, a convolutional layer with multiple filters to transform raw input signals into more expressive latent representations (e.g., the spatial convolution layer in DSSICNN). Such latent representations typically consist of multiple spectral (frequency) channels. Inspired by the phenomenon of CFC, we posit that explicitly modeling the interactions among spectral channels can facilitate the extraction of MI-related discriminative features. However, the majority of existing DL-based MI-EEG decoding approaches largely overlook the modeling of interactions among spectral channels. Given that prior studies have rarely calibrated features according to the relative importance of individual spectral channels, this work designs an attention-based module inspired by CFC to further enhance decoding performance. This module adaptively recalibrates spectral features, thereby promoting the effective representation of cross-spectral interactions. In particular, spectro-wise features are explicitly adjusted via attention mechanisms to capture interactions across spectral bands. Achieving this recalibration necessitates modeling the correlations among distinct frequency components.

To characterize the relationships among spectral bands, global spectro-wise information is initially aggregated. Both max pooling and average pooling are considered effective for capturing critical global spectro-wise features. The computation of this spectro-wise global information is expressed as follows:


$$\begin{array}{l} z_{maxpool}=F_{maxpool}\left(x_{estf}\right) \end{array}$$ (10)

$$\begin{array}{l} z_{avgpool}=F_{avgpool}\left(x_{estf}\right) \end{array}$$ (11)

where $z_{maxpool}\in R^{m}$ is the spectro-wise global information generated by max pooling, *F*_*maxpool*_(·) is the max pooling layer, $z_{avgpool}\in R^{m}$ is the spectro-wise global information generated by average pooling, and *F*_*avgpool*_(·) is the average pooling layer.

Subsequently, the aggregated spectro-wise global information is utilized to systematically capture correlations across spectral bands. The spectro-wise global information obtained in the previous step serves as the initial set of coefficients, which are then employed to model cross-spectral interactions through coefficient reconstruction. Inspired by cross-correlation methods, a procedure is devised for reconstructing these coefficients. The computation for obtaining the reconstructed coefficients is defined as follows:


$$\begin{array}{l} u_{maxpool}\left[i\right]=\overset{-\frac{l}{2}}{\underset{j=\frac{l}{2}}{\sum }}weight_{maxpool}\left[\frac{l}{2}-j\right]z_{maxpool}\left[i-j\right] \end{array}$$ (12)

$$\begin{array}{l} u_{avgpool}\left[i\right]=\overset{-\frac{l}{2}}{\underset{j=\frac{l}{2}}{\sum }}weight_{avgpool}\left[\frac{l}{2}-j\right]z_{avgpool}\left[i-j\right] \end{array}$$ (13)

where $u_{maxpool}\in R^{m}$ and $u_{avgpool}\in R^{m}$ are the spectral context descriptors, $weight_{maxpool}\in R^{l}$ and $weight_{avgpool}\in R^{l}$ are the trainable weight vectors, and *l* is the size of the cross-correlation window.

Once the spectral context descriptors have been obtained, they are integrated through element-wise addition.


$$\begin{array}{l} u=\phi \left(u_{maxpool}+u_{avgpool}\right) \end{array}$$ (14)

where *u*∈*R*^*m*^ is the fused spectral context descriptors, and ϕ(·) is the sigmoid activation function.

Finally, spectro-wise feature recalibration is accomplished by performing element-wise multiplication between the ACSIM input features and the spectral context descriptors. This operation further enhances cross-spectral interactions, as illustrated below:


$$\begin{array}{l} x_{acsif}=u⊗x_{estf} \end{array}$$ (15)

where ⊗ represents the spectro-wise multiplication, and the $x_{acsif}\in R^{m\times t}$ represents the output features of ACSIM.

It is worth noting that several recent studies have begun to introduce attention-based spectral feature recalibration modules. Among them, M-FANet (Qin et al., 2024) is a representative example, which directly employs SENet (Hu et al., 2018) to achieve spectral feature recalibration. However, the two linear layers inherent in SENet, which perform dimensionality reduction followed by dimensionality expansion, may lead to the loss of information in the original spectral channels. In contrast, ACSIM models interactions among spectral channels directly via cross-correlation without relying on fully connected layers, thereby avoiding information loss in the original spectral channels. Moreover, SENet extracts spectral channel information solely through average pooling, whereas ACSIM jointly utilizes average pooling and max pooling, enabling the capture of richer and potentially more informative spectral channel representations.

### 3.4 Attentive spectro-dependent temporal aggregate

Mental fatigue can lead to significant variations in the quality of MI-related temporal fluctuation patterns within the time windows described in Section 3.2.1. This phenomenon occurs because fatigue may compromise a participant's ability to consistently perform MI tasks at certain stages of the experiment. Accordingly, accurately modeling the quality of temporal fluctuation patterns throughout the MI process, in order to assess the contributions of different MI stages to decoding performance, is crucial for enhancing overall decoding accuracy.

To address this challenge, this study proposes a novel attention-based framework that models the quality of MI fluctuation patterns across different stages of MI task execution by subjects, thereby aggregating feature representations from multiple time windows along the temporal axis. This strategy enhances the model's capacity to capture dynamic temporal dependencies. Specifically, attention weights are assigned to quantify the relative significance of features within each time window, and a weighted sum of features across all windows is subsequently computed based on these weights. Furthermore, because the spatial convolution in the Temporal-Spectral-Spatial Branch encodes spectral information within each time window (as detailed in Section 3.2.1), temporal fluctuation patterns may vary across different spectral bands. To account for this variability, independent attention weights are employed to aggregate temporal features for each spectral band individually.

In summary, the computation of ASDTA can be formally expressed as follows:


$$\begin{array}{l} x_{asdtf}\left[i\right]=\overset{t}{\underset{j=1}{\sum }}\alpha_{ij}x_{acsif}\left[i,j\right] \end{array}$$ (16)

$$\begin{array}{l} \alpha_{ij}=\frac{e^{weight_{asdtf}\left[i,j\right]}}{\sum_{j=1}^{t}e^{weight_{asdtf}\left[i,j\right]}} \end{array}$$ (17)

where $x_{asdtf}\in R^{m}$ is the output feature of ASDTA, α_*ij*_ is the attention weight of the *j*^*th*^ time window in the *i*^*th*^ spectral channel, and $weight_{asdtf}\in R^{m\times t}$ is the trainable matrix.

### 3.5 Classification and training

Finally, a linear layer is employed to compute the probability distribution over the classes. The cross-entropy loss function is used to guide the training process. In addition, the Adam optimizer (Kingma and Ba, 2014) is adopted to update the parameters of DSSICNN. The hyperparameters are configured as follows: the number of spatial filters *m* is set to 64, the time window size *w* is set to 250, the dropout rate is set to 0.1, the weight decay is set to 0.001, and the learning rate is set to 0.001.

## 4 Experiments

### 4.1 Datasets

The effectiveness of DSSICNN was evaluated using two publicly available MI-EEG datasets, namely BCIC-IV-2a (Dataset A) (Tangermann et al., 2012) and OpenBMI (Dataset B) (Lee et al., 2019). The MI-EEG data were preprocessed following the methodology described in Liu et al. (2023). No data augmentation was employed to increase the size of the two datasets mentioned above.

#### 4.1.1 BCIC-IV-2a dataset

Dataset A comprises MI-EEG data collected from nine subjects. The MI-BCI paradigm involves four MI tasks: left hand, right hand, tongue, and feet. During data acquisition, signals were recorded from 22 Ag/AgCl electrodes positioned according to the international 10–20 system, with a sampling rate of 250 Hz. The recorded signals were subsequently preprocessed using bandpass filtering between 0.5 Hz and 100 Hz. Each subject participated in two separate sessions conducted on different days, with each session consisting of 288 trials (72 trials per class), and each trial lasting 4s. We directly used the officially provided dataset without performing additional artifact processing, excluding only the three EOG channels. The signals within the 4s interval following cue onset in the entire paradigm were selected as trials. In the officially provided dataset, the left mastoid served as the reference electrode and the right mastoid was used as the ground electrode. No normalization methods were applied.

#### 4.1.2 OpenBMI dataset

Dataset B consists of EEG data collected from 54 subjects. The MI-BCI paradigm comprises two MI tasks: left hand and right hand. EEG signals were recorded using 62 Ag/AgCl electrodes positioned according to the international 10–20 system, with a sampling rate of 1,000 Hz. Each subject participated in two separate sessions conducted on different days. Each session included 200 trials (100 trials per class), with each trial lasting 4s. The recorded MI-EEG signals were subsequently downsampled to 250 Hz. Additionally, following Lee et al. (2019), 20 electrodes located over the motor cortex were selected for analysis (FC-5/3/1/2/4/6, C-5/3/1/z/2/4/6, and CP-5/3/1/z/2/4/6). We directly used the officially provided dataset without performing additional artifact processing, excluding only the four EMG channels. A band-pass filter in the range of 8-30 Hz was applied, and the signals within the 4s interval following cue onset in the entire paradigm were selected as trials. In the officially provided dataset, the nasion served as the reference electrode and AFz was used as the ground electrode. No normalization methods were applied.

### 4.2 Baseline models

DSSICNN was evaluated against a range of baseline models, including Deep ConvNet (Schirrmeister et al., 2017), Shallow ConvNet (Schirrmeister et al., 2017), EEGNet (Lawhern et al., 2018), FBCNet (Mane et al., 2021), SHNN (Liu et al., 2022), EEGConformer (Song et al., 2023), FBMSNet (Liu et al., 2023), IFNet (Wang J. et al., 2023), ADFCNN (Tao et al., 2024), MI-BMInet (Wang et al., 2024), and CLTNet (Gu et al., 2025). To ensure the rigor and fairness of the comparison, most baseline models were implemented using publicly available open-source code.

### 4.3 Experimental settings

To systematically evaluate the performance of DSSICNN, we conducted experiments under both subject-dependent and subject-independent settings. The subject-dependent setting further includes session-dependent and session-independent configurations. Specifically, the subject-dependent setting indicates that both the training and testing sets are drawn from the same subject, whereas the subject-independent setting denotes that the testing set is obtained from a single subject and the training set is constructed from all remaining subjects. The session-dependent setting refers to the case where both the training and testing sets are derived from the first session, with a training-to-testing ratio of 9:1, whereas the session-independent setting indicates that all data from the first session are used as the training set and all data from the second session are used as the testing set.

The Wilcoxon signed-rank test was applied to evaluate the statistical significance of performance differences between DSSICNN and the baseline models. The Wilcoxon signed-rank test is the non-parametric counterpart of the paired t-test. In addition, the Wilcoxon signed-rank test was conducted at the subject level. All models were trained on an NVIDIA Tesla A100 GPU.

## 5 Results and discussion

### 5.1 Comparison of decoding performance

The highest decoding performance is indicated in bold, with * denoting *p* < 0.1 and ** denoting *p* < 0.05. It should be noted that the accuracy of Dataset A refers to the classification accuracy across the four classes.

As presented in Table 1, under the session-independent setting, DSSICNN achieves the highest accuracy and F1-score, attaining 80.09% and 79.79%, respectively, and significantly outperforming most baseline models (*p* < 0.05). Similarly, in the session-dependent setting, DSSICNN records the highest accuracy and F1-score, reaching 86.71% and 86.19%, respectively, significantly surpassing all baseline models. Moreover, in the session-independent setting, DSSICNN outperforms the leading baseline model (ADFCNN) by 2.2% in accuracy and 2.29% in F1-score. In the session-dependent setting, it achieves gains of 4.65% in accuracy and 4.81% in F1-score relative to the best baseline model (FBMSNet). Importantly, DSSICNN delivers these substantial performance improvements while maintaining a parameter-efficient architecture. It is worth noting that CLTNet achieves superior decoding performance compared to DSSICNN. However, CLTNet relies on data augmentation during practical deployment, whereas DSSICNN does not introduce any data augmentation, which to some extent compensates for the slightly lower decoding performance of DSSICNN relative to CLTNet. In addition, CLTNet employs stacked Transformer modules and LSTM modules. The inherent self-attention mechanism within Transformers substantially increases both the spatial and temporal computational complexity of CLTNet, while the intrinsically non-parallelizable computation of LSTM further leads to a significant increase in time complexity. These factors impose certain limitations on the practical deployment of CLTNet in MI-BCI.

**Table 1:** Comparison of decoding performance (average ± std) in % on Dataset A.

**Model**	**Session-independent**		**Session-dependent**	
	**Accuracy**	**F1-score**	**Accuracy**	**F1-score**
CLTNet	83.02 ± 9.50	-	-	-
Shallow ConvNet	66.47 ± 12.60^**^	65.66 ± 12.44^**^	59.63 ± 14.36^**^	57.54 ± 15.01^**^
Deep ConvNet	60.15 ± 18.33^**^	57.51 ± 20.94^**^	57.07 ± 14.16^**^	54.45 ± 14.61^**^
EEGNet	71.10 ± 15.19^**^	70.70 ± 15.33^**^	73.04 ± 14.42^**^	72.56 ± 14.71^**^
FBCNet	74.11 ± 14.70^**^	73.84 ± 14.96^**^	77.56 ± 16.02^**^	77.04 ± 16.47^**^
EEGConformer	71.60 ± 19.55^**^	68.94 ± 23.43^**^	74.63 ± 13.01^**^	72.89 ± 14.49^**^
SHNN	-	-	74.36 ± 14.77^**^	-
FBMSNet	77.28 ± 12.10^*^	76.60 ± 12.68^*^	82.06 ± 11.84^**^	81.38 ± 12.39^**^
IFNet	77.70 ± 14.42^*^	77.11 ± 15.07^**^	81.51 ± 13.88^**^	81.10 ± 14.23^**^
ADFCNN	77.89 ± 10.66^*^	77.50 ± 10.75^*^	79.01 ± 11.27^**^	78.42 ± 11.62^**^
MI-BMInet	77.18 ± 11.52^*^	-	-	-
DSSICNN	80.09 ± 13.72	79.79 ± 14.07	86.71 ± 10.02	86.19 ± 10.57

^*^p < 0.1 and ^**^p < 0.05. Bold values indicate the optimal decoding performance.

The comparative results in Table 2 demonstrate that DSSICNN achieves superior performance in both accuracy and F1-score under session-independent and session-dependent settings on Dataset B. In the session-independent setting, DSSICNN attains an accuracy of 77.87% and an F1-score of 77.54%, significantly outperforming all baseline models (*p* < 0.05). Similarly, in the session-dependent setting, DSSICNN achieves an accuracy of 89.00% and an F1-score of 88.72%, significantly surpassing all baseline models (*p* < 0.05). Furthermore, in the session-independent setting, DSSICNN improves upon the leading baseline model (IFNet) by 4.56% in accuracy and 5.88% in F1-score. In the session-dependent setting, it outperforms the top-performing baseline model (IFNet) by 12.53% in accuracy and 13.39% in F1-score.

**Table 2:** Comparison of decoding performance (average ± std) in % on Dataset B.

**Model**	**Session-independent**		**Session-dependent**	
	**Accuracy**	**F1-score**	**Accuracy**	**F1-score**
Shallow ConvNet	63.96 ± 16.29^**^	62.16 ± 17.65^**^	66.61 ± 16.70^**^	65.27 ± 17.35^**^
Deep ConvNet	67.71 ± 15.27^**^	66.26 ± 16.59^**^	68.67 ± 17.28^**^	67.02 ± 17.97^**^
EEGNet	69.34 ± 16.27^**^	68.01 ± 17.54^**^	70.73 ± 17.46^**^	70.07 ± 17.87^**^
FBCNet	66.72 ± 13.84^**^	64.82 ± 15.75^**^	74.57 ± 13.96^**^	73.64 ± 14.70^**^
EEGConformer	71.14 ± 15.56^**^	68.82 ± 17.77^**^	-	-
FBMSNet	69.27 ± 13.65^**^	67.64 ± 15.14^**^	75.06 ± 14.94^**^	74.03 ± 15.72^**^
IFNet	73.31 ± 15.17^**^	71.66 ± 16.98^**^	76.47 ± 15.57^**^	75.33 ± 16.61^**^
ADFCNN	69.95 ± 13.57^**^	69.43 ± 14.04^**^	73.21 ± 13.96^**^	72.43 ± 14.50^**^
DSSICNN	77.88 ± 12.99	77.56 ± 13.32	89.00 ± 8.53	88.72 ± 8.83

^*^p < 0.1 and ^**^p < 0.05. Bold values indicate the optimal decoding performance.

The outcomes of the comparative experiments indicate that integrating network neuroscience priors into the MI-EEG decoding framework substantially enhances decoding performance relative to conventional CNN and Transformer architectures. Notably, DSSICNN exhibits a more pronounced improvement on Dataset B, which may be attributable to the relatively high incidence of “BCI illiteracy” within this dataset. These findings suggest that DSSICNN is particularly effective in improving decoding performance for individuals with lower inherent BCI proficiency compared with the baseline models.

In addition, as a supplement to the comparison of subject-dependent decoding performance, ADFCNN, FBCNet, EEGNet, Multi-branch 3D CNN (Zhao et al., 2019), CCNN (Amin et al., 2019), CRAM (Zhang et al., 2019) and FBCSPCNN (Bhambare and Jain, 2024) were selected as baselines to evaluate subject-independent decoding performance on Dataset A. The experimental results are presented in Table 3. DSSICNN also achieved the best decoding performance under the subject-independent setting. Its decoding performance was significantly superior to that of most baselines, except for ADFCNN and EEGNet. Moreover, the accuracy and F1-score of DSSICNN exceeded those of the strongest baseline (ADFCNN) by 1.52% and 3.23%, respectively. These results demonstrate that DSSICNN exhibits strong generalization capability and has substantial potential for deployment in MI-BCI. It is worth noting that FBCSPCNN achieves superior decoding performance compared to DSSICNN. However, FBCSPCNN relies on data augmentation in practical deployment, whereas DSSICNN does not introduce any data augmentation, which to some extent compensates for the slightly lower decoding performance of DSSICNN relative to FBCSPCNN.

**Table 3:** Comparison of subject-independent decoding performance (average ± std) in % on Dataset A.

**Model**	**Accuracy**	**F1-score**
FBCSPCNN	92.66	-
CLTNet	58.28 ± 16.56^*^	-
EEGNet	59.74 ± 6.78	58.39 ± 7.54
CRAM	59.10 ± 10.85^*^	-
CCNN	55.35 ± 10.66^**^	-
Multi-branch 3D CNN	52.17 ± 8.76^**^	-
FBCNet	50.25 ± 12.95^**^	46.45 ± 15.32^**^
ADFCNN	61.27 ± 13.07	59.32 ± 15.15
DSSICNN	62.79 ± 15.01	62.55 ± 15.13

^*^p < 0.1 and ^**^p < 0.05. Bold values indicate the optimal decoding performance.

### 5.2 Comparison of efficiency

To further discuss the feasibility of deploying DSSICNN in MI-BCI, Table 4 presents a comparison between DSSICNN and the baselines on Dataset A in terms of the number of trainable parameters, the average training and testing time per epoch, the memory footprint, and the FLOPs. Analysis indicates a strong correlation between the number of trainable parameters and memory footprint: models with a larger number of trainable parameters generally exhibit a larger memory footprint, and vice versa. Based on the comprehensive analysis of Tables 1, 4, DSSICNN achieves a relatively favorable balance among decoding accuracy, spatial complexity, and temporal complexity. Therefore, DSSICNN can be regarded as “parameter-efficient.” Although ADFCNN and IFNet have smaller memory footprints than DSSICNN, DSSICNN exhibits faster training/inference speeds and superior decoding performance compared with both ADFCNN and IFNet. While FBCNet and EEGNet require less memory and achieve faster training/inference than DSSICNN, the decoding performance of DSSICNN is substantially superior to that of FBCNet and EEGNet. Notably, despite having one of the smallest memory footprints, ADFCNN demonstrates the slowest training/inference speeds, which significantly limits its applicability to MI-BCI. We hypothesize that this limitation arises from the inclusion of multi-scale CNN components that cannot be efficiently parallelized, as well as a computationally intensive self-attention mechanism. The FLOPs of DSSICNN are second only to those of EEGNet and EEGConformer. Notably, DSSICNN exhibits higher FLOPs than FBMSNet, whose spatial and temporal complexities are greater than those of DSSICNN. We attribute this difference to the use of spatial convolution layers in DSSICNN with a relatively large number of output channels and full connectivity between input and output channels. In addition, the Spatial Guidance Branch of DSSICNN involves matrix multiplications with relatively high dimensionality, which further increases the FLOPs. Owing to the optimizations for convolution operations and matrix multiplications in the PyTorch framework adopted in this study, DSSICNN is able to maintain favorable training and inference speeds. The number of trainable parameters in FBCSPCNN is substantially higher than that of DSSICNN. This indicates that FBCSPCNN relies, to a certain extent, on data augmentation to expand the training data in order to effectively train a network with a large number of trainable parameters.

**Table 4:** Comparison of efficiency on Dataset A.

**Model**	**Trainable parameters**	**Training/inference time (s)**	**Memory footprint (KB)**	**FLOPs (M)**
EEGNet	4,028	1.03	23	22.93
FBCNet	11,812	1.19	51	7.78
EEGConformer	789,572	5.69	3,125	65.60
FBMSNet	16,267	5.52	72	8.50
IFNet	10,884	4.09	50	2.50
ADFCNN	4,852	9.94	31	2.45
FBCSPCNN	131,025,284	-	-	-
DSSICNN	13,458	3.37	58	12.80

By achieving a favorable balance among decoding accuracy, spatial complexity, and temporal complexity, DSSICNN exhibits strong potential for deployment in MI-BCI. First, prior studies have demonstrated that excessively long recalibration times can reduce user engagement with MI-BCI and thereby degrade MI-BCI performance (Edelman et al., 2025). DSSICNN does not employ data augmentation and thus does not significantly increase data preprocessing time, and it further exhibits favorable training and inference speeds. Consequently, DSSICNN can effectively shorten recalibration time and improve MI-BCI performance. Second, as reported in Forenzo et al. (2025), MI-BCI is often implemented on hardware with limited computational resources, and DSSICNN can effectively reduce hardware memory consumption.

### 5.3 Effect of hyperparameter settings

To evaluate the sensitivity of DSSICNN to hyperparameter selection, experiments were conducted on two datasets under the session-independent setting, investigating the impact of varying hyperparameter configurations on decoding performance.

#### 5.3.1 Effect of the number of spatial filters

**Figure 2:**
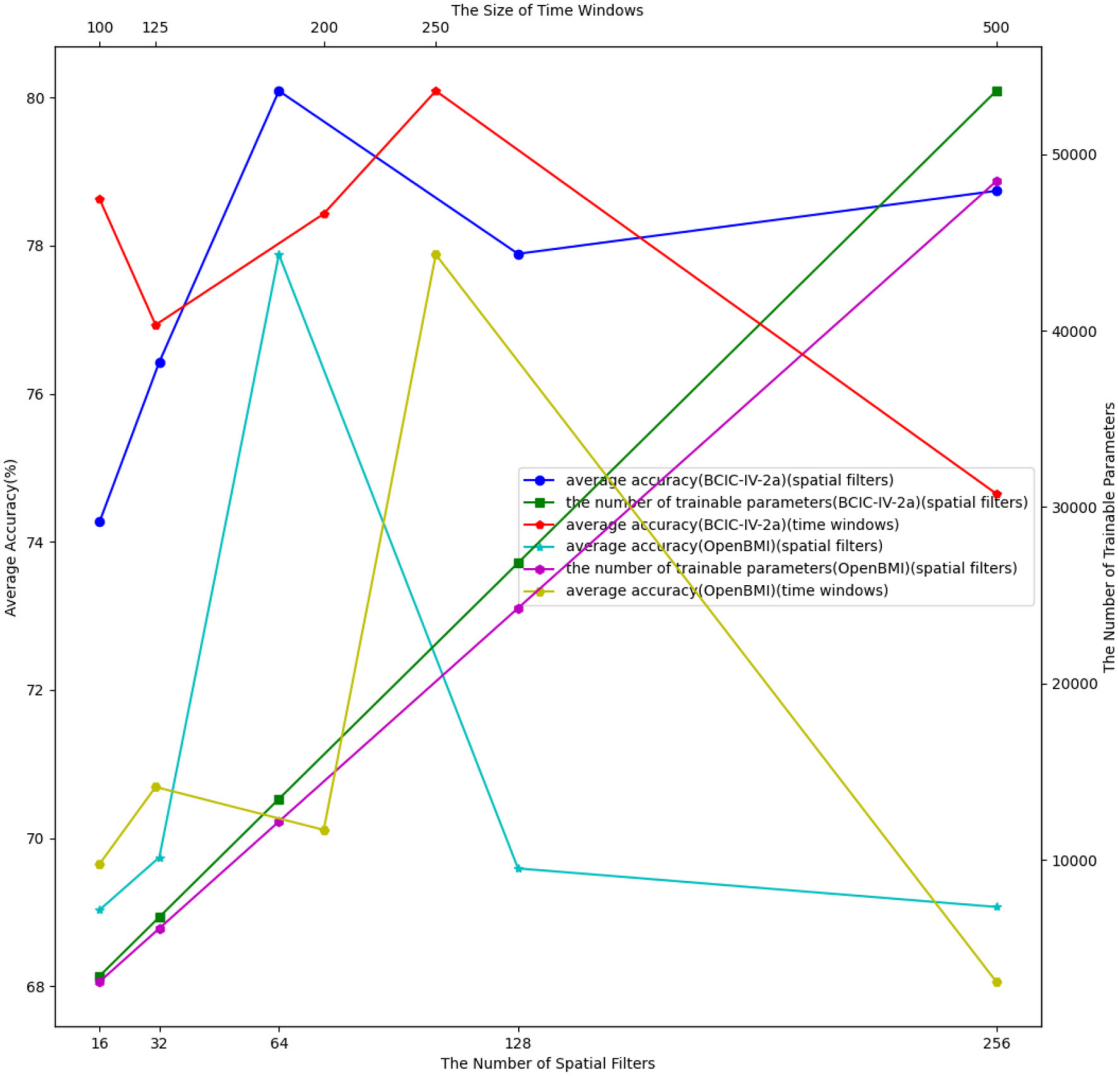
Effect of hyperparameter setting in the session-independent setting.

Figure 2 presents the decoding performance and the corresponding number of trainable parameters under different values of *m*. Decoding performance exhibits notable variability as *m* is adjusted. DSSICNN achieves optimal performance on both datasets when *m* = 64. Additionally, the relationship between the number of trainable parameters and the number of spatial filters follows an approximately linear trend, indicating that the quantity of spatial filters substantially influences the total parameter count. For *m*∈{16, 32, 128, 256}, decoding performance declines to varying degrees, suggesting that neither increasing nor decreasing the number of spatial filters consistently enhances performance. When the number of spatial filters is too small, although computational demands are reduced, the model's generalization capacity is limited, leading to underfitting. Moreover, the number of spatial filters directly impacts the spectral resolution of the subsequent ACSIM module. An insufficient number of spatial filters reduces ACSIM's spectral resolution, resulting in incomplete cross-spectral interactions and diminished decoding accuracy. Conversely, an excessive number of spatial filters compromises the parameter efficiency of DSSICNN, potentially causing overfitting and generating redundant spectral bands. These redundant bands not only increase the number of trainable parameters but may also interfere with effective cross-spectral interactions within ACSIM.

#### 5.3.2 Effect of the size of time windows

As noted in Gabor (1946), an inherent trade-off exists between temporal and frequency resolution in signals, implying that both cannot be simultaneously maximized. Consequently, adjusting the size of the time window alters the amount of temporal information captured, thereby affecting decoding performance. The experimental results shown in Figure 2 indicate that variations in *w* produce substantial fluctuations in decoding accuracy. Optimal performance is observed with *w* = 250 on both datasets, whereas for *w*∈{100, 125, 200, 500}, decoding performance declines markedly. These findings suggest that the relationship between time window size and decoding performance is neither strictly positive nor negative. An excessively small time window diminishes frequency resolution, providing insufficient spectral information for cross-spectral interactions within ACSIM, thereby reducing decoding accuracy. In contrast, an overly large time window inadequately captures fluctuations in subjects' attention during the MI task, failing to appropriately segment periods of focus and distraction, which significantly impairs the effectiveness of ASDTA.

#### 5.3.3 Effect of PLV threshold selection

As outlined in Section 3.1.2, the threshold was initially set to the 100^*th*^ highest PLV value. To examine the influence of threshold selection on decoding performance, experiments were conducted on Dataset A using thresholds corresponding to the 50^*th*^ and 150^*th*^ highest PLV values. The results, summarized in Table 5, indicate that both excessively sparse and overly dense EEG graphs lead to notable performance degradation. In overly sparse graphs, inter-node information flow is severely restricted, and certain nodes may become isolated, preventing the model from effectively capturing interactions among brain regions during the MI task. Conversely, overly dense graphs result in nearly all nodes being interconnected, which, after GNN processing, produces an over-smoothing effect where node features converge to similar values. This redundancy in non-Euclidean spatial information increases the risk of model overfitting. Therefore, the careful selection of the PLV threshold is critical for optimizing decoding performance.

**Table 5:** Comparison of decoding performance under different PLV thresholds (average ± std) in %.

**Rank**	**Accuracy**
50^*th*^	76.39 ± 12.85
100^*th*^	80.09 ± 13.72
150^*th*^	77.12 ± 13.13

### 5.4 Ablation experiments

To evaluate the individual contributions of DSSICNN components to decoding performance, ablation experiments were performed on two datasets under the session-independent setting. The configurations of the DSSICNN variants employed in these studies are detailed in Table 6. In this framework, Branch 1 refers to the Temporal-Spectral-Spatial Branch, while Branch 2 corresponds to the Spatial Guidance Branch.

**Table 6:** Design of DSSICNN variants.

**Model**	**DSIGSTFE**		**ACSIM**	**ASDTA**
	**Branch 1**	**Branch 2**		
DSSICNN-1	✓	✗	✓	✓
DSSICNN-2	✓	✓	✗	✓
DSSICNN-3	✓	✓	✓	✗

**Figure 3:**
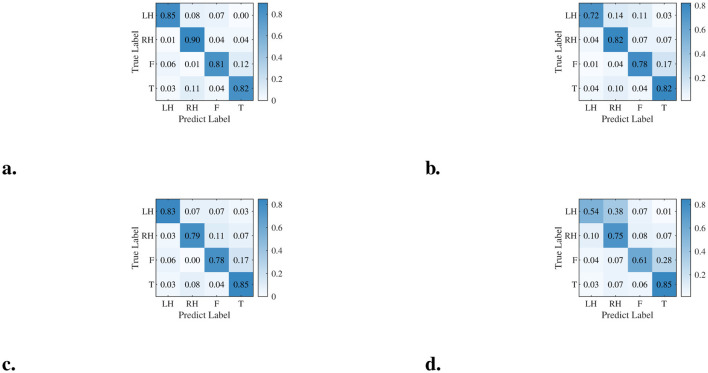
Confusion matrices of DSSICNN and its variants. **(a)** DSSICNN. **(b)** DSSICNN-1. **(c)** DSSICNN-2. **(d)** DSSICNN-3.

The results reported in Table 7 demonstrate that DSSICNN significantly outperforms its variants (*p* < 0.05) across both datasets, underscoring the substantial contributions of each component to overall decoding performance. Figure 3 displays the confusion matrices of DSSICNN and its variants for Subject 3 from Dataset A, showing that DSSICNN consistently achieves higher classification accuracy across all classes. These observations further highlight the essential role of each DSSICNN component in MI-EEG decoding. Examination of the DSSICNN confusion matrix indicates that the foot and tongue classes exhibit the highest misclassification rates across most tasks. This finding aligns with the results reported in Liu et al. (2025) and suggests that DSSICNN still has a limited capacity to extract sufficiently discriminative MI-related features for these two classes.

**Table 7:** Comparison of decoding performance (average ± std) in % between DSSICNN and DSSICNN variants.

**Model**	**Dataset A**		**Dataset B**	
	**Accuracy**	**F1-score**	**Accuracy**	**F1-score**
DSSICNN-1	76.81 ± 14.32^**^	75.99 ± 15.43^**^	70.22 ± 14.39^**^	68.72 ± 15.76^**^
DSSICNN-2	77.39 ± 14.29^**^	76.83 ± 14.92^**^	69.79 ± 14.55^**^	67.67 ± 16.73^**^
DSSICNN-3	73.42 ± 17.44^**^	72.65 ± 18.36^**^	69.74 ± 14.92^**^	67.65 ± 16.88^**^
DSSICNN	80.09 ± 13.72	79.79 ± 14.07	77.88 ± 12.99	77.56 ± 13.32

^*^p < 0.1 and ^**^p < 0.05. Bold values indicate the optimal decoding performance.

For Dataset A, ASDTA emerges as the component exerting the greatest impact on DSSICNN's decoding performance. This effect is likely due to substantial variability in subjects' attention levels during MI task execution, which induces pronounced changes in the temporal fluctuation patterns associated with MI. Further examination of the confusion matrix in Figure 3 elucidates the specific contributions of each DSSICNN component to the classification of distinct MI tasks. In particular, the Spatial Guidance Branch of DSIGSTFE enhances the discriminative capacity of high-level semantic features associated with left-hand movements, whereas ACSIM amplifies the representational power of high-level features related to right-hand movements. In contrast, ASDTA primarily influences decoding accuracy for MI-EEG signals corresponding to regions other than the tongue. Notably, for Dataset B, all DSSICNN components contribute approximately equally to improvements in decoding performance.

### 5.5 Online MI-BCI experiment

**Figure 4:**
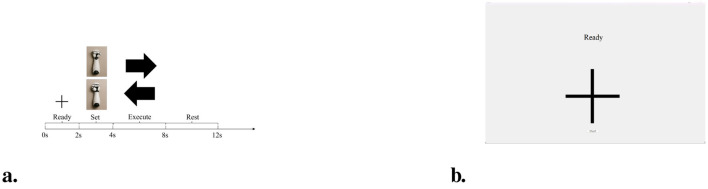
Paradigm and graphical user interface (GUI) of the online MI-BCI experiment **(a)** Paradigm. **(b)** GUI.

To evaluate the feasibility of deploying DSSICNN in real-world applications, and inspired by Forenzo et al. (2024) and Forenzo et al. (2025), a simplified MI-BCI based robotic arm control system was developed in this study. The system comprised three principal components: a signal acquisition module, a laptop computer, and a robotic arm. MI-EEG signals were recorded using the NeuSen W Wireless EEG Acquisition System [Neuracle Technology (Changzhou) Co., Ltd., Changzhou, China] as the signal acquisition module. The laptop was responsible for cue presentation, reception and decoding of the MI-EEG signals transmitted from the acquisition module, and generation of decoding outputs for robotic arm control. The graphical user interface (GUI) and the MI-BCI experimental paradigm are illustrated in Figure 4. The cues presented on the GUI included both textual and graphical instructions, where the textual cues specified the actions to be performed by the participants and the graphical cues indicated the type of MI to be executed. Within the paradigm, the text displayed along the time axis (e.g., “Ready”) corresponded to the textual instructions, whereas the images aligned with the axis represented the graphical instructions. Five participants were recruited for the online experiments, and each participant completed 60 MI trials. The study protocol was approved by the Ethics Committee of Sichuan Provincial Rehabilitation Hospital, China (Approval No. CKLL 2018008), and written informed consent was obtained from all participants. The MI tasks consisted of two classes, left hand and right hand MI, which were mapped to the control of the left and right robotic arms, respectively. A 1s time window was used to segment the subjects' MI-EEG signals as control inputs. Given that the MI-EEG acquisition module operates at a sampling rate of 250 Hz, we set *w* = 50. In addition, the command delivery cadence was reported in terms of the average inter-command interval. For a given subject, a small amount of subject-specific data was first used to calibrate the MI-BCI. Subsequently, online experiments were conducted, and the decoding accuracy, F1-score, and command delivery cadence, reported in terms of the average inter-command interval, were evaluated.

The online experimental results are presented in Table 8. DSSICNN achieved satisfactory decoding performance and command delivery cadence in the online experiments, thereby validating its potential for deployment in MI-BCI.

**Table 8:** Decoding performance in % and command delivery cadence in online experiments.

**Subject**	**Accuracy**	**F1-score**	**Command delivery cadence (s)**
1	75	74.94	6.35
2	80	79.91	6.73
3	70	69.87	5.07
4	87.76	87.72	6.98
5	75	74.99	7.95

### 5.6 Visualization

#### 5.6.1 Spatial distribution of convolutional kernel

**Figure 5:**
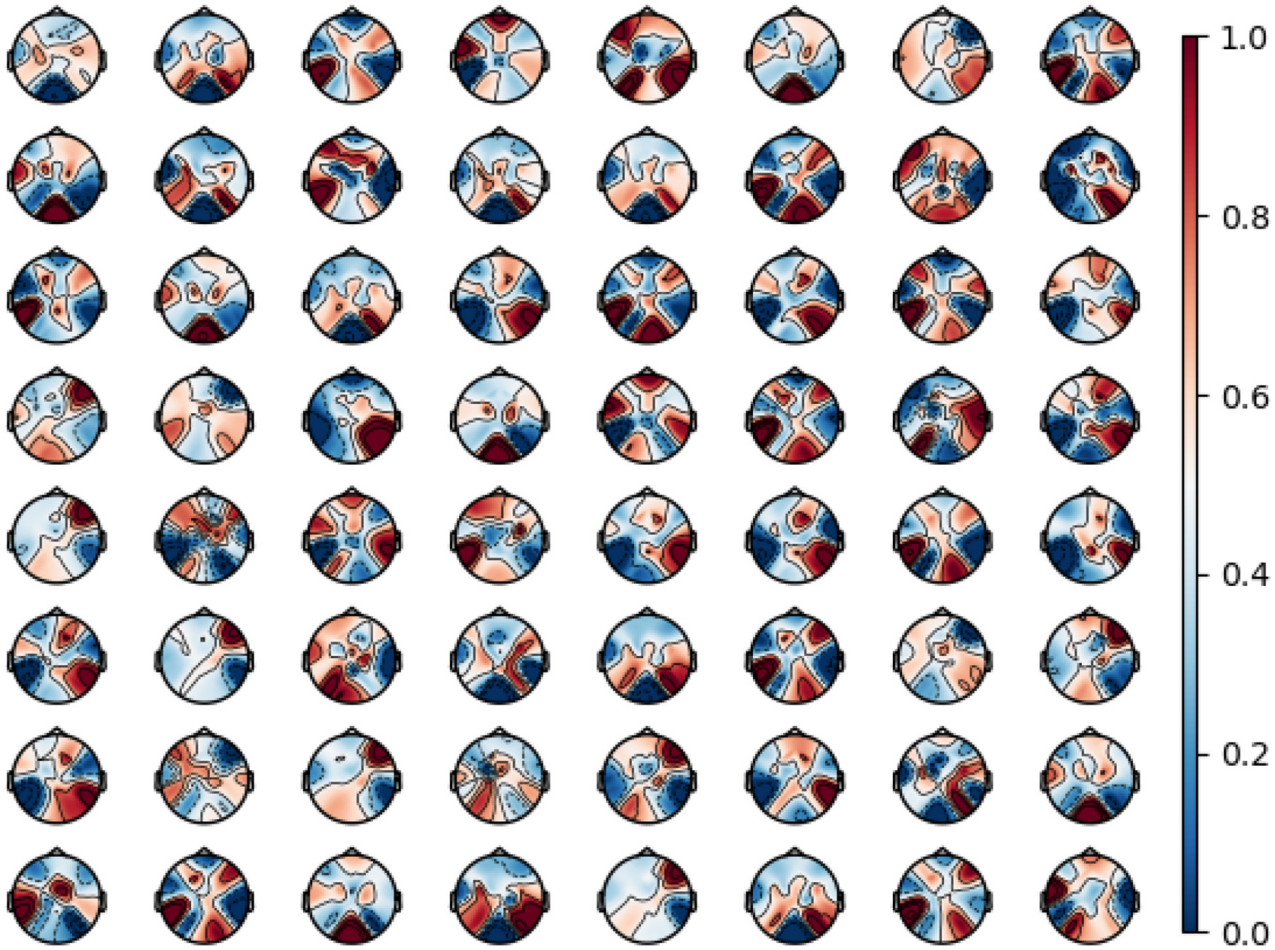
Brain topographies of convolutional kernel weights.

Figure 5 illustrates the spatial distribution of the convolutional kernel weights in DSIGSTFE for Subject 3 of Dataset A. Pronounced variations are observed in the spatial patterns across different spectral bands, indicating that distinct brain regions are selectively engaged for specific spectral components. Additionally, a marked asymmetry is evident in the distribution of kernel weights between the left and right hemispheres, reflecting differential regional activation during various MI tasks and highlighting the hierarchical spatial and functional organization of the brain as a complex network. Notably, the central and parietal regions consistently display the highest kernel weights for Subject 3, corresponding primarily to the motor cortex, which plays a critical role in MI.

#### 5.6.2 Connectivity between brain regions

**Figure 6:**
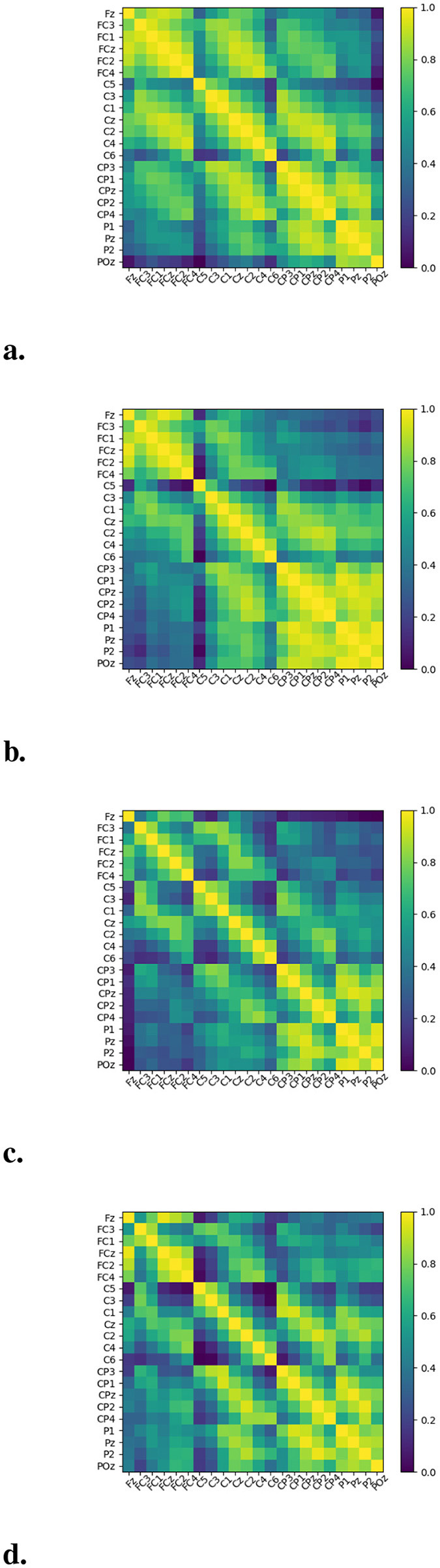
Heatmap of brain regions connectivity strength. **(a)** LH, left hand; **(b)** RH, right hand; **(c)** F, foot; **(d)** T, tongue.

The connectivity strength between brain regions for Subject 3 from Dataset A is visualized using heatmaps, with values normalized to the range 0–1, as shown in Figure 6. Notable variations in connectivity strength are evident across different brain regions during the execution of various MI tasks. These findings suggest that integrating a mechanism to model inter-regional brain connectivity within the MI-EEG decoding framework can enhance the spatial semantic richness of extracted features, thereby improving the framework's capacity for spatial representation.

#### 5.6.3 Importance of various brain regions

In addition to evaluating inter-regional connectivity, we assess the relative importance of individual brain regions to gain deeper insight into the neurophysiological mechanisms underlying MI task execution. Given the hierarchical spatial and functional organization of the brain at the levels of neurons, local circuits, and functional areas (Power et al., 2011), MI tasks facilitate information exchange within specific brain regions. In this context, certain regions act as information hubs during task execution. By conceptualizing the brain as a complex network and representing EEG scalp electrodes over different regions as network nodes, the importance of each brain region can be quantified using node centrality metrics. Node centrality measures the relative significance of nodes within a network. Due to the intrinsic complexity of brain networks, considering only first-order neighborhoods is insufficient. Therefore, eigenvector centrality (Ruhnau, 2000) is employed, as it incorporates the centrality of neighboring nodes, effectively accounting for multi-order neighborhood information. The computation of eigenvector centrality is defined as follows:


$$\begin{array}{c} A\alpha =\lambda_{max}\alpha \end{array}$$ (18)

where *A*∈*R*^*C*×*C*^ is the adjacency matrix, λ_*max*_ is the eigenvalue corresponding to the largest magnitude, and α∈*R*^*C*^ is the eigenvector centrality vector.

**Figure 7:**
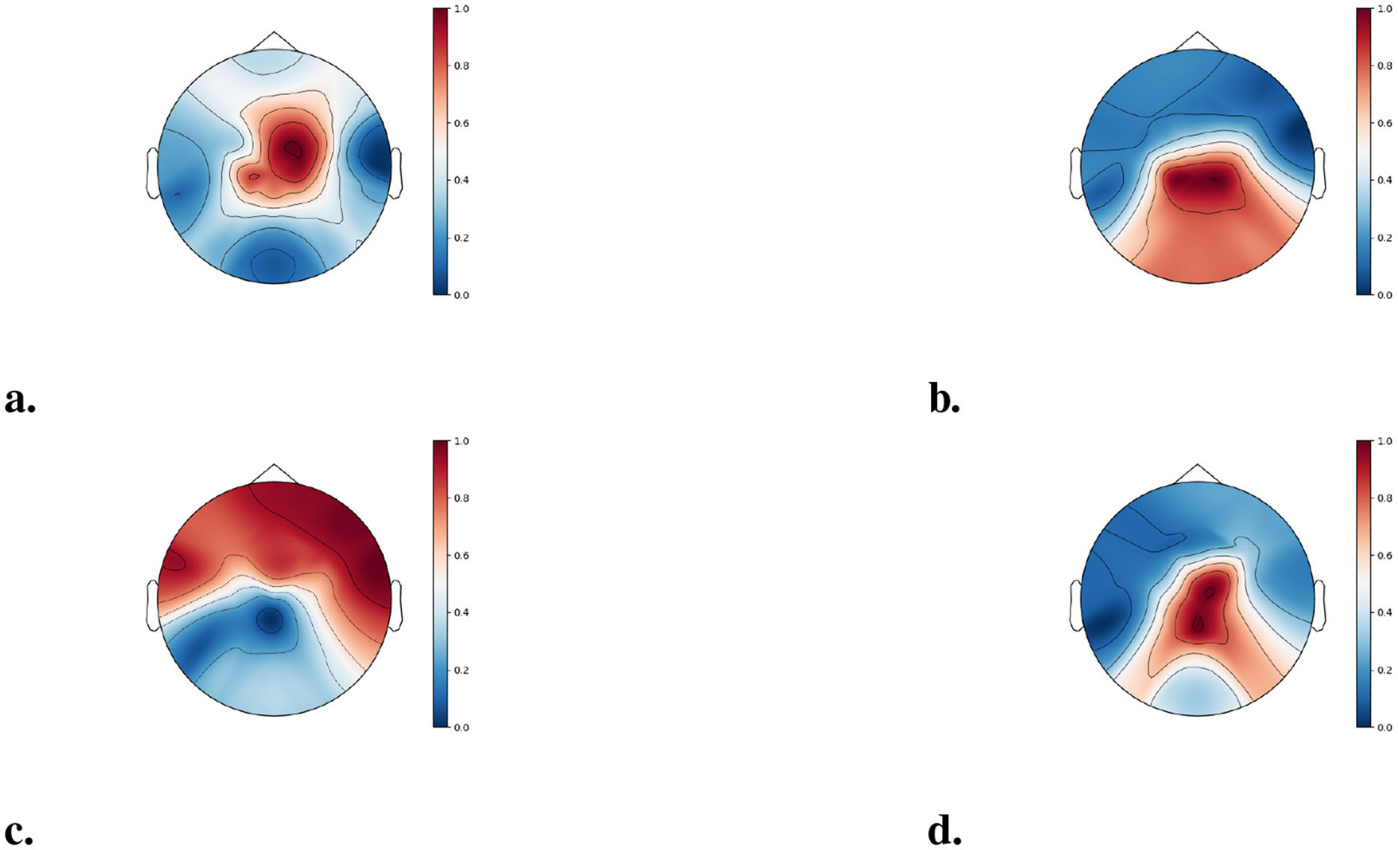
Brain topographies of eigenvector centrality. **(a)** LH, left hand; **(b)** RH, right hand; **(c)** F, foot; **(d)** T, tongue.

The importance of individual brain regions was quantified using eigenvector centrality, and the corresponding brain topographies for Subject 3 from Dataset A are presented in Figure 7. The results indicate that different brain regions display varying levels of engagement, contingent on the specific MI task being performed.

Specifically, during left-hand and tongue MI tasks, a considerable amount of information exchange is localized within the central region, whereas right-hand MI tasks elicit prominent information flow across both the central and parietal lobes. In contrast, foot MI tasks are associated with increased information exchange within the frontal lobe. These findings strongly highlight both the effectiveness and the critical importance of modeling inter-regional information transfer using a dynamic GNN.

#### 5.6.4 Discriminability of high-level semantic feature

**Figure 8:**
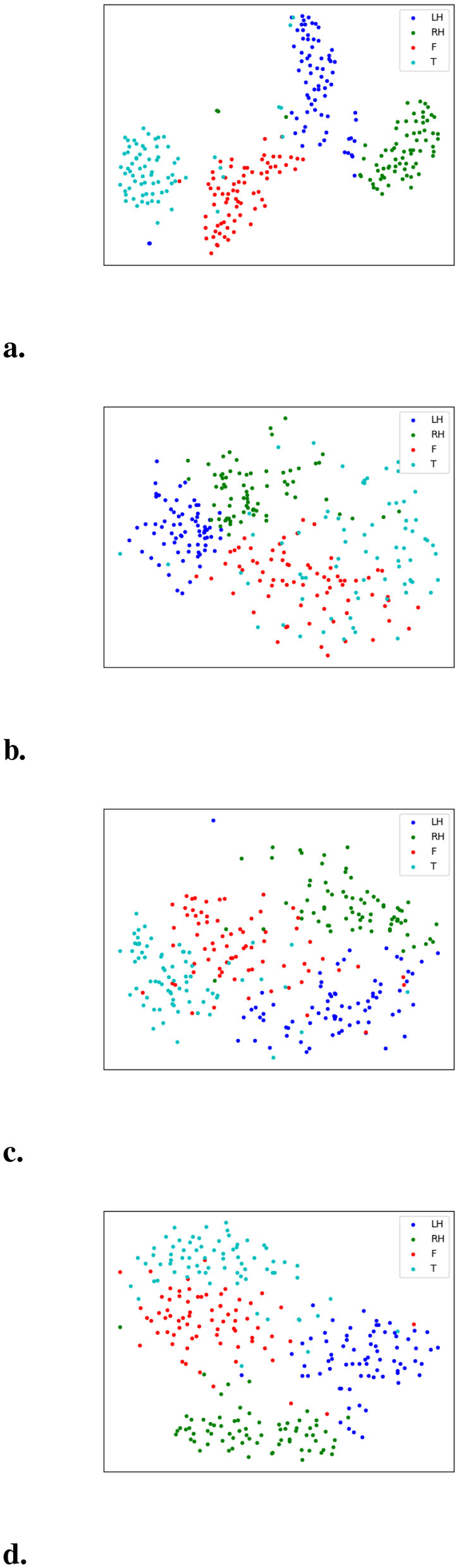
t-SNE of high-level semantic feature distribution (LH: left hand, RH: right hand, F: foot, T: tongue). **(a)** DSSICNN. **(b)** EEGNet. **(c)** FBCNet. **(d)** ADFCNN.

The high-level semantic features, i.e., the representations obtained immediately before the linear classification layer, extracted by DSSICNN and the baseline models were visualized using t-SNE, as shown in Figure 8. The visualizations indicate that the features produced by DSSICNN exhibit markedly greater intra-class compactness and inter-class separability compared with those generated by the baseline models. These findings suggest that DSSICNN effectively captures highly discriminative high-level semantic representations, providing a clear rationale for its superior performance in MI-EEG decoding.

#### 5.6.5 Importance across temporal segments

**Figure 9:**
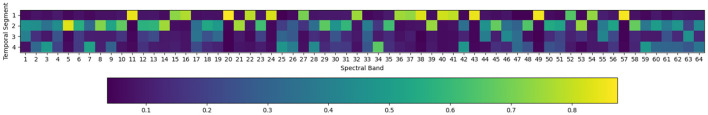
Heatmap of attention weights.

The attention weights assigned to the time windows in ASDTA for Subject 3 from Dataset A are shown in Figure 9. The heatmap reveals pronounced variations in the distribution of attention weights across spectral bands along the temporal dimension. Notably, time windows toward the later stages of the temporal axis generally exhibit higher attention weights, which may reflect the slight temporal overlap between the MI phase and the preceding cue phase in the Dataset A experimental paradigm. Additionally, in several spectral bands, the earliest time window shows markedly elevated attention weights, indicating that these bands are particularly sensitive to spectral information associated with MI preparation.

### 5.7 Limitations and future research

Although the proposed DSSICNN demonstrates excellent decoding performance, several limitations remain. First, while DSSICNN can be effectively trained on small-scale datasets without the need for data augmentation, MI-EEG data are inherently graph-structured, and future work will investigate graph-based data augmentation techniques to increase the effective size of MI-EEG datasets. Additionally, although the brain is modeled as a complex network, the current approach does not explicitly account for its partitioned organizational structure. As a direction for future research, we aim to decompose the whole-brain graph into multiple subgraphs based on established functional brain partitions.

## 6 Conclusion

By incorporating principles from network neuroscience into the MI-EEG decoding framework, we introduce DSSICNN, a model explicitly designed to systematically extract spatial features from MI-EEG signals. The architecture employs a dual-branch parallel design that simultaneously captures spatial information in both Euclidean and non-Euclidean domains. An attention-based mechanism is further integrated to dynamically recalibrate spectro-wise features, thereby enabling effective modeling of cross-spectral interactions. Additionally, the model tracks the evolution of MI-related temporal fluctuation patterns by aggregating feature representations along the temporal dimension. Comparative evaluations on two publicly available datasets demonstrate that DSSICNN significantly outperforms state-of-the-art methods in both session-independent and session-dependent settings. Overall, DSSICNN establishes a novel GNN-based paradigm for MI-EEG decoding and underscores the importance of leveraging the neurophysiological mechanisms underlying MI to enhance decoding performance.

## Data Availability

Publicly available datasets were analyzed in this study. This data can be found here: https://www.bbci.de/competition/iv/#dataset2a; https://gigadb.org/dataset/100542.
